# Comparison of two behavioural pain scales for the assessment of procedural pain: A systematic review

**DOI:** 10.1002/nop2.714

**Published:** 2020-11-28

**Authors:** Hanne Cathrine Birkedal, Marie Hamilton Larsen, Simen A. Steindal, Marianne Trygg Solberg

**Affiliations:** ^1^ Department for Postgraduate Studies Lovisenberg Diaconal University College Oslo Norway; ^2^ Diakonhjemmet Hospital Oslo Norway

**Keywords:** behavioural pain scale, critical‐care pain observation tool, Intensive care, pain assessment, systematic literature review

## Abstract

**Aim:**

To examine the clinical utility and measurement properties of the Critical‐Care Pain Observation Tool and the Behavioural Pain Scale when used to assess pain during procedures in the intensive care unit.

**Design:**

A systematic review was conducted, guided by the Preferred Reporting Items for Systematic Reviews and Meta‐Analyses checklist.

**Methods:**

A systematic search was conducted in CINAHL, MEDLINE, EMBASE and PsychINFO (01 October 2019). Study selection, data extraction and assessment of methodological quality were performed by a pair of authors working independently. Different psychometric properties were addressed: inter‐rater reliability, internal consistency, test–retest reliability, discriminant validity and criterion validity.

**Results:**

Eleven studies were included. Both Critical‐Care Pain Observation Tool and the Behavioural Pain Scale showed good reliability and validity and were good options for assessing pain during painful procedures with intensive care unit patients unable to self‐report on pain. The Critical‐Care Pain Observation Tool is to be preferred since this tool was shown to have particularly good reliability and validity in assessing pain during procedures, but the Behavioural Pain Scale is an appropriate alternative.

## 1 INTRODUCTION

Critically ill patients experience frequent pain and discomfort throughout their stay in the intensive care unit (ICU) and pain seems to be the patients’ worst memory after discharge (Gélinas, 2007; Zetterlund et al., 2012). Uncontrolled pain has significant short‐ and long‐term psychological and physiological consequences, delaying recovery and even being life‐threatening (Baron et al., 2015; Barr et al., 2013; Peng et al., 2014; Puntillo et al., 2014). Treatment in the ICU may be provided while the patient is already under stress, such as the fear of losing his/her life or the threat of not regaining well‐being (Gélinas, 2016). This more affective dimension of pain was emphasised in a recent proposal to change the definition of pain, which now reinforces pain as a distressing experience associated with actual or potential tissue damage and with sensory, emotional, cognitive and social components (Williams & Craig, 2016).

A recent concept analysis stated discomfort as physical or psychological, characterized by unpleasant feelings resulting in avoidance or reduction of the source of the discomfort (Ashkenazy & Ganz, 2019). The study concluded that pain is one of the sources for discomfort, but not every discomfort can be attributed to pain. Hence, clinicians may interpret discomfort as pain, when the patient, in reality, is uncomfortable. This may be especially true in the ICU with non‐communicative patients, making accurate assessments of pain imperative for correct treatment. Day‐to‐day nursing procedures and interventions can potentially be a considerable source of pain or discomfort for ICU patients. The fact that more than 30% of ICU patients regardless of diagnosis experience pain at rest and that this percentage exceeds 50% during common care procedures, underscores the need for high‐quality pain management (Chanques et al., 2006; Gélinas, 2007; Puntillo et al., 2014). Some of the most painful procedures experienced by ICU patients are nursing care procedures such as turning, endotracheal suctioning, tube and drain removal, wound care and arterial line insertion (Payen et al., 2007; Puntillo et al., 2014; Vázquez et al., 2011).

Systematic pain assessment with valid tools is essential for adequate pain management and acts as an indicator of good clinical practice (Skrobik et al., 2010; Wøien & Bjørk, 2013). The patient's self‐report of pain is regarded as the gold standard in the assessment of pain (Breivik et al., 2008; Merskey, 2007). However, in the ICU, a number of patients are unable to self‐report and verbally communicate their pain and discomfort due to critical illness, decreased level of consciousness, mechanical ventilation and sedation. This makes pain assessment and pain management even more challenging (Alderson & McKechnie, 2013; Chanques et al., 2006; Payen et al., 2009). Therefore, for an assessment of pain, observable behavioural and physiological indicators become important indices (Gélinas et al., 2006).

## 2 BACKGROUND

There are numerous tools for assessing pain in adult ICU patients, including the Nonverbal Pain scale (NVP), the Critical‐Care Pain Observation Tool (CPOT), the Behavioural Pain Scale (BPS), the Comfort scale, the Face, Legs, Activity, Cry, Consolability scale (FLACC), all of which have numeric rating scales (Rose et al., 2013). Of all these tools, the CPOT and the BPS are the most commonly used (Aïssaoui et al., 2005; Rijkenberg et al., 2015). They seem valid and sensitive for capturing changes in pain response among patients receiving sedatives or lacking the ability to communicate (Ahlers et al., 2008; Barr et al., 2013; Gélinas, 2007; Young et al., 2006). In two systematic reviews (Gelinas et al., 2013; Pudas‐Tähkä et al., 2009) that compared the psychometric properties of pain assessment scores for intensive care patients who were unable to self‐report pain, the CPOT and the BPS received the best quality assessment scores. The CPOT was designed to detect pain in critically ill patients (Gélinas et al., 2006), while the BPS was developed to assess pain in unconscious mechanically ventilated patients (Payen et al., 2001). The main difference between these tools is in their evaluation of body movements and muscle tension (Severgnini et al., 2016). Improved pain management is associated with better outcomes for ICU patients (Chanques et al., 2006; Payen et al., 2009; Robinson et al., 2008; Skrobik et al., 2010). However, pain caused by procedures in the ICU appears to remain underestimated and undertreated (Puntillo et al., 2014; Siffleet et al., 2007).

To ensure that the measurement error of pain assessment tools is as small as possible, the tools’ validity and reliability need to be determined to ensure the instruments are functioning correctly (Field, 2013). Validity refers to whether the instrument measures what it is intended to measure (Polit & Beck, 2013) and reliability is the ability of the pain assessment tool to deliver the same results under the same circumstances (Field, 2013).

Several systematic reviews have compared a number of pain assessment scales used in the ICU (Barzanji et al., 2019; Fischer et al., 2019; Grosso et al., 2019; Pudas‐Tähkä et al., 2009). The aim of these studies was for example to identify the most used pain assessment scales for the critically ill unconscious adult patient (Grosso et al., 2019) and instruments developed for pain assessment in unconscious or sedated intensive care patients (Pudas‐Tähkä et al., 2009). Furthermore, for a pain scale to guide pain management decisions and to support efficient evaluations, it must be actionable and easy to interpret and it cannot take so many resources that it disrupts clinical care in the hectic ICU context. A feasible, useful and accurate scale is essential to ensure that the pain of ICU patients is correctly and consistently identified by procedures. However, to our knowledge, no reviews have evaluated studies that use both the CPOT and the BPS in relation to procedures in the ICU with the purpose of informing and guiding nurse decision‐making. This systematic review therefore aimed to examine the measurement properties of the CPOT and BPS when used to assess pain during procedures in the ICU. It was directed by the following research questions:

- To what extent have the CPOT and the BPS been tested for validity, reliability and responsiveness during painful procedures in the intensive care setting?
- Which of these two tools is best suited to assess pain in non‐verbal critically ill intubated patients during painful procedures?

## 3 THE STUDY

### 3.1 Design

This systematic review was conducted according to the Preferred Reporting Items for Systematic Reviews and Meta‐Analyses (PRISMA) checklist (Moher et al., 2009). The protocol was not published or registered.

### 3.2 Method

#### 3.2.1 Eligibility criteria

Studies were included if they met the following criteria: 1) they had a quantitative design; 2) they included ICU patients aged 18 years or older who were unable to self‐report pain due to critical illness; 3) the patients received mechanical ventilation and/or sedation; and 4) were tested for the validity and reliability of both the CPOT and the BPS during painful procedures. Studies were excluded if the data were published as a conference paper, abstract, doctoral thesis, letter or comments.

#### 3.2.2 Search strategy

A systematic literature search was conducted on 01 October 2019, using the databases CINAHL, MEDLINE, EMBASE and PsychINFO. The search strategy was built in MEDLINE by two of the authors and an experienced librarian, using text words and Medical Subject Heading. This search was adapted to the other databases. In the databases, the only limitation used was language restricted to Danish, English, Norwegian and Swedish caused by our available language knowledge. Searches were performed without restriction on publication year and ended on 01 October 2019. The Medline search strategy is described in Data S1.

#### 3.2.3 Search outcomes

Our primary outcomes were the validity and reliability of the CPOT scale and the BPS scale. The CPOT scale includes four behavioural indicators: 1) facial expression; 2) body movements; 3) muscle tension; and 4) compliance with the ventilator (for intubated patients) or verbalization (for extubated patients) (Gélinas et al., 2006). The BPS scale includes three behavioural indicators: 1) facial expression; 2) movements of the upper extremities; and 3) compliance with the ventilator (Payen et al., 2001).

#### 3.2.4 Study selection and data extraction

**Figure 1:**
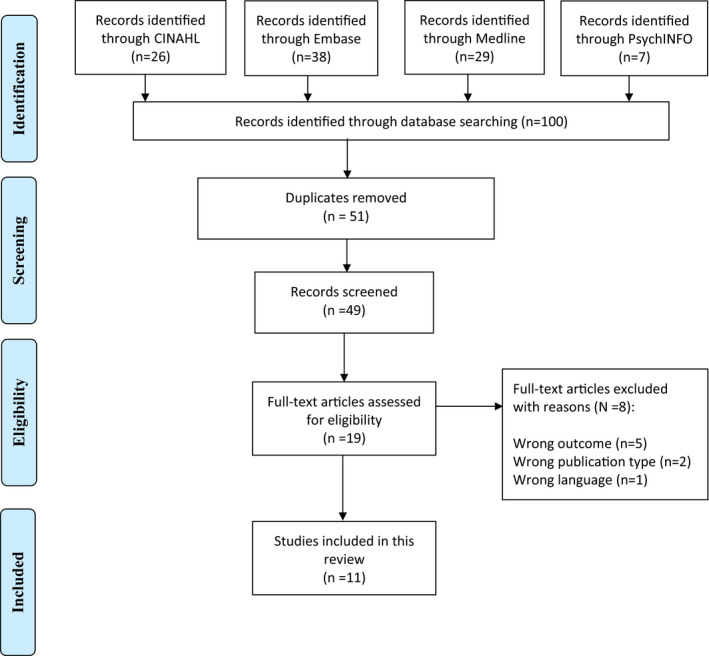
PRISMA 2009 flow diagram

A pair of authors independently assessed whether titles, abstracts and full‐text papers met the inclusion criteria. When there was any doubt whether a paper should be included, a third author independently assessed the paper. The data from the included papers were extracted independently by the same pair of authors using a standardized data collection form that included: author, year, location for research, aim, study design, population and results. Reasons for excluded articles are presented in Figure 1.

#### 3.2.5 Quality assessment

The methodological quality of the included studies was assessed by the pair of authors independently, using the Critical Appraisal Skill Programs (CASP) checklist (Critical Appraisal Skills Programme, 2018; Nadelson & Nadelson, 2014). The quality assessment criteria for the included articles are shown in Table S1.

### 3.3 Analysis

To assess the validity and reliability of the CPOT and BPS pain assessment tools, the results from the studies included were organized according to psychometric properties, such as inter‐rater reliability, internal consistency, test–retest reliability, discriminant validity and criterion validity (see Table S2). Due to heterogeneity in study design, patient population, intervention, context and time of pain assessment, a quantitative synthesis was not possible. Consequently, the results are presented in a narrative form and with a table describing the validity and reliability scores and the analyses of each paper.

## 4 RESULTS

The literature search identified 100 publications. After removal of duplicates, 51 titles and abstracts were screened. After this first screening, 32 articles were excluded as they did not meet the inclusion criteria. The full text of 19 papers was assessed, and the final sample included a total of 11 studies: Nine prospective observational studies, one crossover observational study and one cross‐sectional study (Figure 1). No studies were identified that employed randomized controlled trial designs. The studies were conducted in the USA (*N* = 1), Taiwan (*N* = 2), Saudi Arabia (*N* = 1), China (*N* = 1), Iran (*N* = 1), Brazil (*N* = 1), Finland (*N* = 1), the Netherlands (*N* = 2) and Italy (*N* = 1). The sample sizes ranged from six–316 ICU patients. The painful procedures were endotracheal suctioning (*N* = 8), turning (*N* = 5) and standardized nociceptive stimulation by pressure algometry (*N* = 1). The characteristics of the included studies are shown in Table 1.

**Table 1:** Characteristics of the included studies

Author/ year/ Country	Aim	Study design	Population	Painful procedures	Quality appraisal CASP
Al Darwish et al. (2016) Saudi Arabia	To find the most reliable, sensitive and valid tool for assessing pain	Descriptive research design, Cohort	MV ICU patients (*N* = 47)	Endotracheal suctioning	Y:11 C:3 N:0
Chanques et al. (2014) USA	To compare psychometric properties of CPOT and BPS	Observational study	Non‐intubated and intubated medical ICU patients (43% lightly sedated, 57% with delirium, 63% MV (*N* = 30)	Repositioning Turning Endotracheal suctioning Mobilisation	Y:13 C:1 N:0
Cheng et al. (2018) Taiwan	To compare validity of Chinese versions of CPOT and BPS	Crossover observational study	Conscious or unconscious MV ICU patients (*N* = 316)	Endotracheal suctioning	Y:11 C:3 N:0
Gomarverdi et al. (2019) Iran	To compare the severity of pain measured by BPS and CPOT during procedures	Cross‐sectional study	Mechanically ventilated ICU patients (*N* = 90)	Endotracheal suctioning	Y:10 C:4 N:0
Hsiung et al. (2016) Taiwan	To evaluate a translation of BPS and CPOT in the traditional Chinese language spoken in Taiwan	Prospective observational study (pilot)	Unconscious and/or sedated and MV ICU patients (*N* = 10)	Endotracheal suctioning	Y:10 C:3 N:1
Klein et al. (2018) Brazil	To translate the English versions of the BPS and CPOT into Brazilian Portuguese and to validate their use in ICU	Prospective cohort study	MV and non‐MV medical‐surgical ICU patients (*N* = 168)	Exposed to 1) Standardized nociceptive stimulation by pressure algometry (SNSPA) 2) Standard care (turning)	Y:12 C:1 N:1
Liu et al. (2015) China	To assess and compare the reliability and validity of Chinese versions of CPOT and BPS	Prospective observational study	Non‐intubated and intubated general ICU patients (*N* = 117)	Endotracheal suctioning	Y:13 C:1 N:0
Pudas‐Tähkä & Salanterä (2018) Finland	To test the reliability of three Finnish translations of the pain assessment tools	Prospective observational study	MV and sedated ICU patients (*N* = 6)	Endotracheal suctioning	Y:8 C:6 N:0
Rijkenberg et al. (2015) Netherlands	To compare the discriminant validation and reliability of CPOT and BPS in order to find the most useful clinical pain assessment tool	Prospective observational cohort study	MV medical ICU patients (*N* = 68)	Turning of the patient	Y:12 C:1 N:1
Rijkenberg et al. (2017) Netherlands	To compare inter‐rater reliability, internal consistency and discriminant validation of BPS and CPOT after cardiac surgery	Prospective, observational cohort study	MV ICU patients after cardiac surgery (*N* = 72)	Turning of the patient	Y:12 C:1 N:1
Severgnini et al. (2016) Italy	To compare CPOT and BPS separately	Prospective observational study	Conscious and unconscious ill MV ICU patients (*N* = 101)	Turning Endotracheal suctioning	Y:11 C:2 N:1

Abbreviations: BPS, Behaviour Pain Scale; C, Can't tell; CPOT, Critical‐care Pain Observation Tool; MV, mechanically ventilated; N, No; Y, Yes.

### 4.1 Quality assessment

A summary of the assessments of methodological quality is shown in Table 1 and in Table S1. Overall, the quality of the articles was rated as relatively high and 10 of the 11 articles presented with a score that had more than 10 out of a possible 14 “Yes” assessments. The assessments did show that the question of whether the outcomes were accurately measured to minimize bias was not sufficiently reported on in the articles. Furthermore, the question “How precise are the results?” was difficult to assess, as very few of the studies provided confidence intervals for their mean values, which could have given a more precise estimate of the range in which the real answers lay.

### 4.2 Reliability

Four studies calculated weighted κ coefficients as a measure of inter‐rater reliability (Chanques et al., 2014; Cheng et al., 2018; Klein et al., 2018; Liu et al., 2015). Chanques et al. (2014) measured 0.81 for both tools and Liu et al. (2015) showed that both showed nearly perfect reliability (BPS: 0.94; CPOT: 0.98). Cheng et al. (2018) measured four times and got greater variation (BPS: 0.73–0.80; CPOT: 0.64–1.00) but also showed relatively high reliability. Liu et al. (2015) showed that the inter‐rater reliability was not significantly different with the intubated compared with the non‐intubated patients when using the CPOT (0.985 and 0.955). The BPS had a significantly greater inter‐rater reliability for non‐intubated compared with intubated patients (0.939; 0.977, respectively).

Four studies (Al Darwish et al., 2016; Pudas‐Tahka & Salantera, 2018; Rijkenberg et al., 2015, 2017) calculated inter‐rater reliability using the intraclass correlation coefficient (ICC). Rijkenberg et al. (2015) and Rijkenberg et al. (2017) calculated the ICC between the CPOT and the BPS, showing a substantial score for all assessments (0.75; 0.74; 0.74; 0.62), respectively (see Table 2). Pudas‐Tähkä et al. (2018) used the Shrout‐Fleiss ICC test during suctioning and showed that the best results following the painful procedure showed slightly lower scores for the BPS than for the CPOT. Al Darwish et al. (2016) showed the lowest agreement in the Facial Expression subscale during suction when using the BPS (*r* = .77), while in the CPOT, they found weak agreement in the Muscle Tension subscale.

**Table 2:** Reliability and Validity findings of BPS and CPOT during painful procedures in non‐verbal critically ill intubated patients

Research study	Reliability findings			Validity findings	
	Inter‐rater reliability	Internal consistency	Test–retest reliability of BPS and CPOT	Discriminant validity	Criterion Validity
Al Darwish et al. (2016)	ICC: BPS: Lowest agreement in the Facial Expression subscale during suctioning, *r* = .77 CPOT: Weak agreement in the Muscle Tension subscale during suctioning, *r* = .47	Cronbach's α: BPS: 0.95 CPOT: 0.95 Pearson's correlation: BPS: 0.90 CPOT: 0.93	NR	Effect size coefficient BPS: 1.2 CPOT: 1.37	NR
Chanques et al. (2014)	Weighted‐κappa: BPS: 0.81 (± 0.03) CPOT: 0.81 (± 0.03)	Cronbach's α: BPS: 0.81 CPOT: 0.80 *p* = .48	NR	No median significant differences between observations at baseline and after procedure BPS: *p* = .41 CPOT: *p* = .74 Effect size coefficient (Mann–Whitney Wilcoxon test): BPS: 1.99 CPOT: 1.55	NR
Cheng et al. (2018)	Weighted‐κappa assessed four times: BPS: 0.73–0.80 CPOT: 0.64–1.00	NR	NR	ANOVA Significant differences in mean score from rest to suction BPS: *p* < .01 CPOT: *p* < .01	MLRA found that CPOT and BPS (T1‐T2) were significantly associated to self‐reported pain *p* < .05
Gomarverdi et al. (2019)	NR	NR	NR	Spearman correlation during suction: BPS: median 7 (Q1 = 5, Q3 = 8) CPOT: median 4 (Q1 = 2, Q3 = 5) *r* = .88, *p* < .001 Wilcoxon coefficient: BPS from rest to suction *Z* = −8.05, *p* < .001 CPOT from rest to suction, *Z* = −8.01 *p* < .001	NR
Hsiung et al. (2016)	NR	Cronbach's α: BPS: 0.74 CPOT: 0.70	NR	From rest to suction: BPS: Median increased from 4–5 in 100% of patients CPOT: Median increased from 1–2 in 90% of patients	NR
Klein et al. (2018)	Weighted‐κ during turning: BPS: 0.94 (95% CI 0.92–0.95) CPOT: 0.96 (95% CI 0.94–0.97) *p* < .001	NR	NR	Kendall's W showed significant increase in median score of pain from rest to turning BPS: 0.92 *p* < .05 CPOT: 0.93 *p* < .05	NR
Liu et al. (2015)	Weighted‐κ: BPS intubated: 0.939 BPS non‐intubated: 0.977 CPOT intubated: 0.985 CPOT non‐intubated: 0.955	Cronbach's α: BPS intubated: 0.78 BPS non‐intubated: 0.81 CPOT intubated: 0.79 CPOT non‐intubated: 0.81	NR	Mann–Whitney test: BPS: *Z* = −14.468, CPOT: *Z* = −14.183 *p* < .001, respectively	Spearman correlation: *r* = .951, *p* < .001
Pudas‐Tähkä & Salanterä (2018)	The Shrout Fleiss ICC test during suctioning BPS: 0.29 CPOT: 0.18	Cronbach's α after suction (at rest) BPS: 0.86 CPOT: 0.96	Bland–Altman plot: Retest values within one point for both measures (95% CI)	NR	NR
Rijkenberg et al. (2015)	ICC: BPS: 0.74 (95% CI 0.68–0.79) *p* = .001 CPOT: 0.075 (95% CI, 0,69–0,79) *p* = .001	Cronbach α: BPS: 0.70 CPOT: 0.71	NR	Friedman test, at rest to painful procedure: BPS: 3.0 (3.0–3.0) to 5.0 (4.0–6.0), *p* < .001 CPOT: 0.0 (0.0–0.0) to 2.0 (0.0–3.0), *p* = .002	NR
Rijkenberg et al. (2017)	ICC at turning BPS: (95% CI) 0.748 (0.637–0.836) CPOT: (95% CI) 0.622 (0.456–0.746)	Cronbach α: *Nurse 1* BPS: 0.62 CPOT: 0,65 *Nurse 2* BPS: 0.59 CPOT:0,58	Substantial agreement 0,74 (95% CI, 0,68–0,79) *p* = .001 for all measurements for both scales	Friedman test, at rest to painful procedure. *Nurse 1:* BPS: 3.0 (3.0–4.0) to 5.0 (4.0–6.0), *p* = .001 CPOT: 0.0 (0.0–0.0) to 2.0 (0.0–3.0), *p*= .001 *Nurse 2:* BPS: 3.0 (3.0–4.0) to 5.0 (4.0–6.0) *p* = .001 CPOT: 0.0 (0.0–0.0) to 2.0 (0.3–3.0) *p* = .001	NR
Severgnini et al. (2016)	NR	NR	NR	Wilcoxon coefficient: During care in unconscious patients: BPS: *Z *= −10.68, *p* = <0.001 CPOT: *Z *= −10.62, *p* = <0.001 During care in conscious patients: BPS: *Z *= −6.93, *p* = <0.001 CPOT: *Z *= −6.48, *p* = <0.001 Movement comparisons between scales (Cohen's κ): During κ = 0.64 Effect size: 1.4	During nursing care BPS: Sensitivity 62.8% BPS: Specificity 91.7% CPOT: Sensitivity 76.5% CPOT: Specificity 70.8%

Abbreviations: CI, confidence interval; ICC, intraclass correlation coefficient; MLRA; Multiple logistic regression analysis; NR, not reported; weighted‐κ, weighted kappa coefficients.

Seven studies calculated internal consistency using Cronbach's alpha (Al Darwish et al., 2016; Chanques et al., 2014; Hsiung et al., 2016; Liu et al., 2015; Pudas‐Tahka & Salantera, 2018; Rijkenberg et al., 2015, 2017). All these studies, except for Rijkenberg et al. (2017), showed that the CPOT and BPS had satisfactory to good internal consistency. Chanques et al. (2014) showed good internal consistency for both the CPOT and the BPS (0.81; 0.80, respectively). These authors found no significant difference between the CPOT and the BPS (*p* = .48), and there was no significant difference in Cronbach's alpha coefficients between intubated and non‐intubated patients (0.82; 0.81 and 0.81; 0.83, respectively). However, Pudas‐Tähkä and Salanterä (2018) showed that Cronbach's alpha values varied greatly with both instruments. The lowest values were recorded for those measurement points where the pain scores were 0. The highest scores were achieved after endotracheal suctioning at rest (Table 2). Rijkenberg et al. (2015) indicated that the CPOT and the BPS both had acceptable internal consistency during a painful procedure (0.71; 0.70, respectively). Liu et al. (2015) showed satisfactory values for both the CPOT and the BPS, but with higher scores in the non‐intubated patients than in the intubated. In this study, the values showed good internal consistency for both tools (intubated = 0.785; 0.981; non‐intubated = 0.812; 0.812, respectively). Al Darwish et al. (2016) presented the best results with a Cronbach's alpha for internal consistency of 0.95 in both scales.

### 4.3 Validity

Validity was assessed using discriminant validity in ten studies (Table 2) and three studies also reported on criterion validity by using different analyses (Cheng et al., 2018; Liu et al., 2015; Severgnini et al., 2016). Only one study did not report on any validity tests (Pudas‐Tahka & Salantera, 2018). About discriminant validity, Chanques et al. (2014) tested the responsiveness of the CPOT and the BPS using an effect size coefficient and demonstrated a significantly higher responsiveness of the BPS compared with the CPOT (1.99 and 1.55, respectively). Al Darwish et al. (2016) also used the effect size coefficient and here the CPOT scored 1.37, while the BPS scored 1.20.

Rijkenberg et al. (2015) and Rijkenberg et al. (2017) calculated the discriminant validity between the CPOT and the BPS using the Friedman test. In Rijkenberg et al. (2015), the median scores increased by two points from rest to painful procedure (*p* < .001). The median BPS scores between rest and non‐painful procedure showed a significant increase of one point (*p* = .002), whereas the median CPOT score remained unchanged. In Rijkenberg et al. (2017), the median CPOT and BPS scores for both nurses increased significantly (*p* = .001) from rest to painful procedure (Table 2).

Klein et al. (2018) showed a significant increase in the median scores for pain in both measures between rest and turning, using Kendall's W; here, the BPS scored 0.92 and the CPOT 0.93. Cheng et al. (2018) also showed significant differences in the mean scores from rest to suction by using ANOVA. In the pilot study by Hsiung et al. (2016), a total of 100% of patients showed an increased score for the BPS between rest and suction compared with 90% of the patients for the CPOT.

Liu et al. (2015) calculated the discriminant validity using the Mann–Whitney test. The scores for the CPOT and the BPS during painful procedures were significantly higher than during non‐painful procedures (*p* < .001). There was no significant difference between the scores at rest and during non‐painful procedures (*p* > .05).

Severgnini et al. (2016) calculated discriminant validity using the Wilcoxon coefficient for the CPOT and the BPS during care with conscious and unconscious patients. Both tools showed a statistical difference during nursing care (*p* < .0001) and after nursing care (*p* < .0001). They also compared the two tools at three different times by using the Cohen's kappa before (κ = 0.69), during (κ = 0.64) and after nursing care (κ = 0.66). Furthermore, they evaluated criterion validity by using Spearman rho (ρ) and by comparing the two pain scales using a VAS scale. Strong correlations with VAS were found, which included all measurements (ρ = 0,48 and ρ = 0.56). Gomarverdi et al. (2019) used a similar approach by using the Spearman correlation during suction. Here, BPS had a median value of 7, while CPOT had a median value of 4, with a strong correlation (*r* = .88, *p* < .001). The Wilcoxon coefficient was highly significant for both measures from rest to suction (CPOT: *Z *= −0.8.01 and BPS: *Z *= −8.05).

## 5 DISCUSSION

This systematic review aimed to examine the measurement properties of the CPOT and BPS when used to assess pain during procedures in the ICU. Due to clinical and methodological heterogeneity across the studies included, a quantitative synthesis was not possible. Several methodological limitations, including pre‐experimental design approaches, limited control of confounders and small sample sizes, burden the body of evidence. The main findings are that the CPOT and the BPS both show good reliability and validity and are both good options for assessing painful procedures in the ICU. However, certain issues were identified in the studies, involving both pain assessment tools.

This review showed that inter‐rater reliability showed that the nurses assessing the pain had a substantial to near perfect agreement in their observations related to the measurement of pain in mechanically ventilated ICU patients. However, the BPS showed a significantly greater inter‐rater reliability in non‐intubated compared with intubated patients, which may indicate that the BPS needs further assessment in intubated patients for nurses to provide adequate pain management to this latter group of patients (Chanques et al., 2014; Liu et al., 2015). The most likely reason for the BPS having a higher score in the non‐intubated patients is the fact that BPS requires assessing ventilator waveform and asynchrony, which could be difficult while simultaneously observing a patient's face and body. Listening to ventilator alarms, as used by the CPOT, could be a useful alternative and CPOT may therefore be a more accurate tool for assessing pain in intubated patients (Chanques et al., 2014; Liu et al., 2015). However, systematic assessment for pain in mechanically ventilated ICU patients at rest and 30 min after any procedure resulted in smaller doses of sedation being required, a three day reduced duration on the respirator and a five‐day reduction in ICU stay (Payen et al., 2009).

Most studies included measured internal consistency by estimating Cronbach's alpha and the results for both tools mostly showed satisfactory to good internal consistency. This indicates that the correlation between the behavioural domains is sufficient and can be considered reliable for measuring pain during painful procedures. This finding is consistent with the systematic review by Barzanji et al. (2019) that evaluated pain assessment tools in non‐verbal intubated critically ill adult patients after open heart surgery. Here, they found satisfactory Cronbach alpha values for both CPOT and BPS, indicating moderate to high internal reliability. Nevertheless, Rijkenberg et al. (2017) found insufficient Cronbach's alpha values in their study and they indicated that higher values from other studies should be interpreted with caution due to missing calculations for complete sample sizes.

The results about discriminant validity suggest that both pain assessment tools were well suited to measure the presence of pain when moving from rest to a painful procedure. However, there were some concerns about the BPS as it also showed a significant increase in scores during non‐painful oral care, while the CPOT score remained unchanged (Rijkenberg et al., 2015, 2017). These studies reported that most of the increase in BPS score during oral care was the result of changes in facial expression and movements of the upper limbs. The increase might have been due to reflexes to touch rather than response to pain. Coughing and straining might also be reflexes due to movement of the endotracheal tube during oral care (Rijkenberg et al., 2017). The difference in discriminant validation of the CPOT and BPS during the non‐painful stimulus could also be the result of the different numbers of scoring options in each domain. For the BPS, nurses have to choose between four different scoring options compared with three scoring options for the CPOT. It is possible that the four scoring options of the BPS are less clearly distinguished than the three scoring options of the CPOT and could therefore lead to incorrect assessment of a non‐painful stimulus.

Severgnini et al. (2016) showed that both CPOT and BPS scores increased during nursing care in both unconscious and conscious patients. In conscious patients, during nursing care, the BPS showed higher specificity and lower sensitivity than the CPOT. Different individual items are included in the BPS and CPOT. Muscular tone and movement of arms and legs are included in the CPOT but not in the BPS (Severgnini et al., 2016). Scores may differ due to the “muscle tension” item of the CPOT, an item not included in the BPS. For patients with high muscle tension related to pain, the CPOT would be a more effective assessment tool (Liu et al., 2015). Facial expression and ventilator compliance are recorded in both scales, although using different individual scores. Severgnini et al. (2016) showed that facial expression was the most important parameter related to pain assessment. It is important to note that facial expression is also easier to score at the bedside. A limitation in the study by Severgnini was that discriminant validity should be assessed during both painful and non‐painful procedures in the same population. If the values calculated through the tools are increased by both painful and non‐painful procedures, the validity and reliability are questionable.

The results suggest that both the CPOT and the BPS are reliable and valid pain assessment tools. However, the CPOT seems to be the preferred option for assessing pain during painful procedures due to its discriminant validation, meaning that CPOT can better detect pain whenever the patient is believed to be in pain. This may also be an important tool to distinct between discomfort and pain to provide the best treatment (Ashkenazy & Ganz, 2019). On the other hand, the BPS is rated as a little easier to remember during clinic practice than the CPOT as the BPS has only three domains for observation rather than four domains, as included in the CPOT (Chanques et al., 2014).

### 5.1 Limitations

There are limitations to our systematic review that need to be addressed. The systematic literature search was limited to the English and Scandinavian languages and publication types such as conference papers, abstracts, doctoral theses, letters and comments were excluded. Consequently, the results may be affected by publication bias. However, we searched multiple databases and collaborated with a librarian to ensure that the search was extensive. Furthermore, owing to the pre‐experimental, pre‐test–post‐test nature of the designs, several threats to validity are potentially present, involving selection bias, lack of blinding, the order in which the instruments were tested and cultural competence. For example, in the study by Rijkenberg et al. (2015), the nursing staff were not blinded and when pain assessments were performed, the assessors were aware of which procedures were to be performed. This may have led them to perceive more behavioural changes during events, leading to higher scores during painful procedures. Additionally, the BPS was always completed first. An essential consideration is that no gold standard has been established for pain assessment in patients who are unable to give self‐reports.

## 6 CONCLUSION

Both of the pain assessment tools addressed in this review have a systematic approach to evaluating pain. The CPOT especially has been shown to have good reliability and validity for assessing pain during painful procedures in ICU patients unable to self‐report their pain. The BPS is an appropriate alternative, but because of the discriminant validation, the CPOT is to be preferred.

## CONFLICTS OF INTEREST

We have no conflict of interest.

## AUTHOR CONTRIBUTIONS

HCB and MTS: Manuscript drafting, conception and design. HB, MHL, SAS and MTS: Acquisition of data, analysis and interpretation. All authors: Manuscript revision and final approval of manuscript to publish in Nursing Open.

## ETHICAL APPROVAL

No approval from the university college or the data protection officer was needed to conduct the review since we investigated already published data.

## Supporting information

Supplementary Material

Supplementary Material

Supplementary Material

## Data Availability

Data availability is not relevant, since all data are available in original articles.
