# Supplementary material for: Comparison of two behavioural pain scales for the assessment of procedural pain: A systematic review
Source: Nurs Open. 2020 Nov 28;8(5):2050–60. doi: 10.1002/nop2.714 (PMC8363347; doi:10.1002/nop2.714)
Supplement: Supplementary file 1 — Supplementary Material [file NOP2-8-2050-s003.docx]

Supplementary file 1

| Database(s): Ovid MEDLINE(R) and In-Process & Other Non-Indexed Citations 1946 to September 30, 2019 | | |
| --- | --- | --- |
| Search Strategy: | |  |
| **#** | **Searches** | **Results** |
| 1 | (critical adj3 (pain adj2 observation*)).tw. | 103 |
| 2 | cpot.tw. | 87 |
| 3 | 1 or 2 | 116 |
| 4 | (behavioral adj2 (pain adj2 scale)).tw. | 160 |
| 5 | bps.tw. | 6093 |
| 6 | 4 or 5 | 6197 |
| 7 | 3 and 6 | 33 |
| 8 | limit 7 to (danish or english or norwegian or swedish) | 29 |
