# Supplementary material for: Comparison of two behavioural pain scales for the assessment of procedural pain: A systematic review
Source: Nurs Open. 2020 Nov 28;8(5):2050–60. doi: 10.1002/nop2.714 (PMC8363347; doi:10.1002/nop2.714)
Supplement: Supplementary file 2 — Supplementary Material [file NOP2-8-2050-s001.docx]

# Supplementry file 2

# Quality appraisal by using Critical Appraisal Skills Program (CASP)

| CASP COHORT | Did the study address a clearly focused issue? | Was the cohort recruited in an acceptable way? | Was the exposure accurately measured to minimise bias? | Was the outcome accurately measured to minimise bias? | Have the authors identified all important confounding factors? | Have they taken account of the confounding factors in the design and / or analysis? | Was the follow up of subjects complete enough? | Was the follow up of subjects long enough? | What are the results of this study? | How precise are the results? | Do you believe the results? | Can the results be applied to the local population? | Do the results of this study fit with other available evidence? | What are the implications of this study for practice? |
| --- | --- | --- | --- | --- | --- | --- | --- | --- | --- | --- | --- | --- | --- | --- |
| Al Darwish et al. (2016) | Y | Y | Y | Y | C | C | Y | Y | Y | C | Y | Y | Y | Y |
| Chanques et al. (2014) | Y | Y | Y | Y | Y | Y | C | Y | Y | Y | Y | Y | Y | Y |
| Cheng et al. (2018) | Y | Y | Y | Y | C | Y | Y | Y | Y | C | Y | Y | Y | C |
| Gomarverdi et al (2019) | Y | Y | Y | C | C | C | Y | Y | Y | C | Y | Y | Y | Y |
| Hsiung et al. (2016) | Y | Y | Y | N | C | C | Y | Y | Y | C | Y | Y | Y | Y |
| Klein (2018) | Y | Y | Y | N | Y | Y | Y | Y | Y | C | Y | Y | Y | Y |
| Liu et al. (2015) | Y | Y | Y | Y | Y | Y | Y | Y | Y | C | Y | Y | Y | Y |
| Pudas (2018) | Y | C | Y | C | C | C | Y | Y | Y | C | C | Y | Y | Y |
| Rijkenberg, (2015) | Y | Y | Y | N | Y | Y | Y | Y | Y | C | Y | Y | Y | Y |
| Rijkenberg, (2017) | Y | Y | Y | N | Y | Y | Y | Y | Y | C | Y | Y | Y | Y |
| Severgnini (2016) | Y | Y | Y | N | Y | C | Y | Y | Y | C | Y | Y | Y | Y |

Y,Yes; C,Can’t tell; N,No
