# Supplementary material for: Comparison of two behavioural pain scales for the assessment of procedural pain: A systematic review
Source: Nurs Open. 2020 Nov 28;8(5):2050–60. doi: 10.1002/nop2.714 (PMC8363347; doi:10.1002/nop2.714)
Supplement: Supplementary file 3 — Supplementary Material [file NOP2-8-2050-s002.docx]

### Supplementary file 3

### Statistical tests to describe the reliability and validity methods used in the included articles

| Psychometric  property | Description | Coefficient or  analysis | Level of  acceptability |
| --- | --- | --- | --- |
| Interrater reliability | The degree to which two raters operating independently, agree on their observations/ratings (of the CPOT and the BPS) of a phenomen, which in this case is the measurement of pain. | Weighted-κ  Z-test  ICC | >0.80= Near perfect  >0.60= Important  >0.40= Moderate agreement  (Landis & Koch, 1977)  0.21-0.40= Fair agreement  0.41-0.60= Moderate  0.61-0.80= Substantial  >0.80= Almost perfect  (Landis & Koch, 1977) |
| Internal consistency | The degree to which the subparts of an instrument are measuring the same attribute or dimension, as a measure of the instrument`s reliability. | Cronbach α | >0.70= Satisfactory  >0.80= Good  (Streiner, Norman & Cairney, 2015) |
| Test-retest reliability | Assessment of the stability of an instrument by correlating the scores obtained on two administrations. | Spearman correlation coefficient |  |
| Discriminant validity | Refers to the ability of an instrument to measure the presence or the absence of the variable, and if the intervention makes a difference. | Wilcoxon coefficient  Friedman test  Post hoc analysis: Wilcoxon signed rank test  Effect size | <0.20= Small  <0.50= Moderate  >0.80= Large  (Polit & Beck, 2013) |
| Criterion Validity | The ability of an instrument to accurately measure the phenomen of interest, in this case, measurement of pain. | Spearman rank correlation |  |
